# The role of height-associated loci identified in genome wide association studies in the determination of pediatric stature

**DOI:** 10.1186/1471-2350-11-96

**Published:** 2010-06-14

**Authors:** Jianhua Zhao, Mingyao Li, Jonathan P Bradfield, Haitao Zhang, Frank D Mentch, Kai Wang, Patrick M Sleiman, Cecilia E Kim, Joseph T Glessner, Cuiping Hou, Brendan J Keating, Kelly A Thomas, Maria L Garris, Sandra Deliard, Edward C Frackelton, F George Otieno, Rosetta M Chiavacci, Robert I Berkowitz, Hakon Hakonarson, Struan FA Grant

**Affiliations:** 1 Division of Human Genetics, The Children's Hospital of Philadelphia, Philadelphia, Pennsylvania 19104, USA; 2 Department of Biostatistics and Epidemiology, University of Pennsylvania, Philadelphia, Pennsylvania 19104, USA; 3 Center for Applied Genomics, Abramson Research Center, The Children's Hospital of Philadelphia, Philadelphia, Pennsylvania 19104, USA; 4 Behavioral Health Center and Department of Child and Adolescent Psychiatry, The Children's Hospital of Philadelphia, Philadelphia PA 19104, USA; 5 Center for Weight and Eating Disorders, Department of Psychiatry, University of Pennsylvania, Philadelphia PA 19104, USA; 6 Department of Pediatrics, University of Pennsylvania, Philadelphia PA 19104, USA

## Abstract

**Background:**

Human height is considered highly heritable and correlated with certain disorders, such as type 2 diabetes and cancer. Despite environmental influences, genetic factors are known to play an important role in stature determination. A number of genetic determinants of adult height have already been established through genome wide association studies.

**Methods:**

To examine 51 single nucleotide polymorphisms (SNPs) corresponding to the 46 previously reported genomic loci for height in 8,184 European American children with height measurements. We leveraged genotyping data from our ongoing GWA study of height variation in children in order to query the 51 SNPs in this pediatric cohort.

**Results:**

Sixteen of these SNPs yielded at least nominally significant association to height, representing fifteen different loci including *EFEMP1-PNPT1, GPR126, C6orf173, SPAG17*, Histone class 1, HLA class III and *GDF5-UQCC*. Other loci revealed no evidence for association, including *HMGA1 and HMGA2*. For the 16 associated variants, the genotype score explained 1.64% of the total variation for height z-score.

**Conclusion:**

Among 46 loci that have been reported to associate with adult height to date, at least 15 also contribute to the determination of height in childhood.

## Background

Height has been correlated with various disorders, including the observations that taller people are at a higher risk of developing cancer and shorter people are more likely to present with type 2 diabetes [1-3]. Determination of height in humans has long been considered to be largely influenced by genetic factors; indeed, twin and family studies have suggested that as much as 90% of variation in human height is genetically determined[4-8].

For many years, studies have attempted to identify genetic factors influencing human height in order to provide insights into human growth and development. Prior to 2007, genome-wide linkage and candidate-gene association studies had limited success in this regard; however, with the recent emergence of genome wide association (GWA) studies, tens of common genetics variants influencing height have now been uncovered, primarily in adults[9-14].

Weedon *et al *published the first GWA study of height using the Affymetrix GeneChip Human Mapping 500 K platform on nearly 5,000 individuals of self-reported European ancestry[9]. As a consequence, they observed association to common variation in the mobility group-A2 (*HMGA2*) oncogene. Follow-up analyses in approximately 19,000 more individuals (both adults and children) revealed strong replication of this observation. A subsequent GWA study uncovered another height locus, *GDF5-UQCC*, using data from the FUSION and SardiNIA cohorts[10].

These initial discoveries were followed by four meta-analyses with larger sample sizes, which collectively revealed 44 additional height loci [11-14]. However, some lack of overlap between the results of these GWA studies has been observed, which may be partly explained by the different statistical powers of the studies[15].

Although the causal variants at these loci have still to be elucidated, it has been shown that many of the implicated genes are involved in pathways influencing bone and cartilage development, including skeletal development signaling (*PTCH1, HHIP, BMPs, GDF5*), the extracellular matrix (*ACAN, FBLN5, EFEMP1, ADAMTS17, ADAMTSL3*), chromatin structure and regulation (*DOT1L, SCMH1, HMGA2*) and cell cycle regulation and mitosis (*CDK6, ANAPC13, NCAPG*)[15]. In addition, some of the loci were novel and are now a clear focus of attention in height biology.

In this study we aimed at examining these initial and meta-analysis findings that were previously reported to be genome wide significant in a large European American pediatric cohort with height measurements to determine the relative impact of these variants on childhood stature. For this purpose, we leveraged genotyping data from our ongoing GWA study of height variation in children.

## Methods

### Study population

All subjects were consecutively recruited from the Greater Philadelphia area from 2006 to 2009 at the Children's Hospital of Philadelphia and its Primary Care Centers. Our study cohort consisted of 8,184 children of European ancestry with height information. All subjects were biologically unrelated and were aged between 0 and 18 years old. The basic characteristics of the study subjects are outlined in Table 1. This study was approved by the Institutional Review Board of the Children's Hospital of Philadelphia. Parental informed consent was given for each study participant for both the blood collection and subsequent genotyping.

**Table 1:** Basic characteristics of the study subjects, including sample size and mean height plus standard deviation (S.D.) for each age and gender separately

	MALE			FEMALE		
**Age**	**N**	**Average Height (cm)**	**S.D**.	**N**	**Average Height (cm)**	**S.D**.

Under 2	673	73.51	10.33	424	73.04	8.86
2	319	88.92	5.79	200	87.87	6.40
3	318	97.72	5.50	244	96.17	5.58
4	279	104.22	5.97	183	103.66	6.30
5	215	110.75	7.05	175	110.40	6.88
6	218	119.01	7.23	177	117.98	6.52
7	219	125.14	7.42	159	124.19	7.23
8	197	130.01	7.81	157	128.87	7.56
9	196	135.56	8.20	145	133.52	9.99
10	184	139.98	8.73	177	139.64	8.62
11	188	145.59	9.39	188	147.47	9.06
12	220	150.60	10.64	181	152.92	9.17
13	221	157.79	10.28	243	157.21	8.40
14	237	164.66	9.40	248	160.25	7.25
15	252	169.09	9.48	260	161.39	7.69
16	201	172.90	7.90	275	162.64	6.77
17	171	174.38	8.52	216	162.89	7.29
18	113	174.60	7.01	111	163.47	7.58

### Genotyping

We performed high throughput genome-wide SNP genotyping using either the Illumina Infinium™ II HumanHap550 or Human 610 BeadChip technology in the same manner as our center has reported previously[16]. The SNPs analyzed survived the filtering of the genome wide dataset for SNPs with call rates < 95%, minor allele frequency < 1%, missing rate per person < 2% and Hardy-Weinberg equilibrium *P *< 10^-5^.

Loci described from GWA studies published to date have been found using either the Affymetrix or Illumina platform. In the event a locus was reported using both the Illumina and Affymetrix arrays, we used the SNPs present on the Illumina array. In the event of a signal only being described on the Affymetrix array, we either already had that SNP on our Illumina array or we identified and used the best surrogate SNP available (see Additional file 1: Supplemental Table S1 for the surrogates employed).

### Statistical analyses

From our database of heights for our multi-dimensional scaling (MDS) determined Caucasians, as previously described[17-19] and resulting in a low genomic inflation factor, we eliminated height outliers using 2% cutoff for each age category in order to remove potential measurement error. As height values vary widely across pediatric age groups and gender, we calculated the Z-scores using inverse-normal transformation for each age (one year bin) and gender category, and conducted association analysis with the Z-scores as the outcome variable.

We queried the data for the indicated SNPs in our pediatric samples. All statistical analyses were carried out using the software package *plink*[20]. By treating the Z-score for height as a quantitative trait, association analysis for each SNP was carried out using linear regression with the SNP included as an independent variable (coded as 0, 1, and 2, counting the number of minor alleles at the SNP).

**Figure 1:**
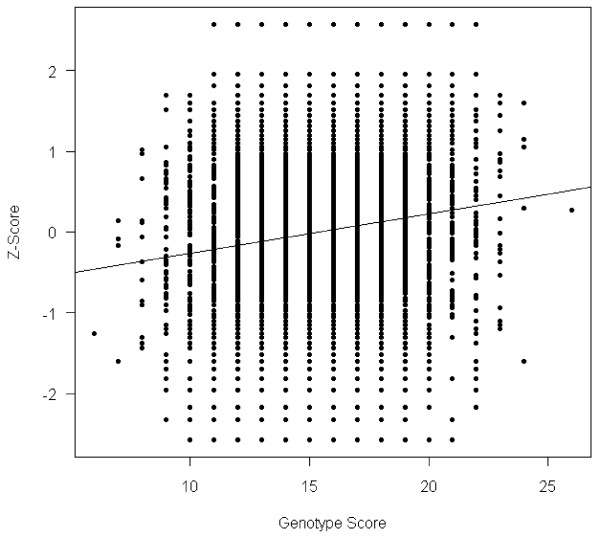
**Scatter plot for association between height z-score and the genotype score by summing the number of height increasing alleles across all 16 height-associated SNPs**.

The results for Figure 1 were generated by summing the number of height increasing alleles across all 16 height-associated SNPs in our study to in order to produce a scatter plot showing the impact of the genotype score on the cumulative height Z-score.

## Results

The 51 SNPs corresponding to the 46 previously reported height loci were investigated with respect to their association to normalized pediatric height in MDS-determined European Americans (Table 2; also Additional file 2: Supplemental Table S2 for analyses by age categories).

**Table 2:** Quantitative association results for the candidate loci in the European American height cohort (n = 8,184), sorted by chromosomal location.

Chr	Minor Allele	SNP	Position (Build 36)	Nearby genes(s)	NMISS	MAF	BETA	SE	R2	T	P
1	A	rs11809207	26205282	*CATSPER4*	8106	0.1730	0.02775	0.0213	0.0002095	1.303	0.1926
1	C	rs6663565	41232781	*SCMH1*	8184	0.4297	0.03744	0.01587	0.0006802	2.36	0.0183
**1**	**C**	**rs17038164**	**118574711**	***SPAG17***	**8182**	**0.2601**	**-0.06029**	**0.01784**	**0.001395**	**-3.38**	**0.0007274**
1	G	rs11205277	146705945	Histone class 2A, *MTMR11, SV2A, SF3B4*	8182	0.4195	0.0109	0.01579	5.83E-05	0.6906	0.4898
1	G	rs678962	168921546	*DNM3*	8178	0.2183	0.01272	0.0192	5.37E-05	0.6625	0.5077
1	A	rs2274432	180752602	*C1orf19, GLT25D2*	7965	0.3237	0.04568	0.01722	0.0008828	2.653	0.008003
1	A	rs3942992	224079131	*ZNF678*	8181	0.1725	9.96E-05	0.02072	2.83E-09	0.004809	0.9962
**2**	**G**	**rs3791679**	**56008543**	***EFEMP1, PNPT1***	**8179**	**0.2539**	**-0.0782**	**0.01798**	**0.002308**	**-4.349**	**1.39×10^-5^**
2	T	rs1052483	219759853	*IHH, CRYBA2, FEV, SLC23A3, TUBA1*	8110	0.0956	-0.04701	0.02686	0.0003777	-1.75	0.08009
3	C	rs9841212	135674636	*ANAPC13, CEP63*	8154	0.3289	-0.006868	0.01676	2.06E-05	-0.4098	0.682
3	A	rs6763931	142585531	*ZBTB38*	8174	0.4091	0.04634	0.01587	0.001042	2.92	0.003513
4	T	rs6842303	17530324	*LCORL, NCAPG*	8173	0.2466	0.02231	0.01823	0.0001834	1.224	0.2209
4	C	rs6830062	17693999	*LCORL, NCAPG*	8184	0.1883	-0.05215	0.02014	0.0008192	-2.59	0.009613
4	A	rs1812175	145932449	*HHIP*	8172	0.1639	-0.03329	0.02125	0.0003002	-1.566	0.1173
5	T	rs10472828	32924575	*NPR3*	8182	0.4585	-0.005366	0.01589	1.39E-05	-0.3376	0.7357
6	A	rs12198986	7665058	*BMP6*	8183	0.4516	-0.0003072	0.01598	4.52E-08	-0.01922	0.9847
**6**	**G**	**rs10946808**	**26341366**	**Histone class 1, Butyrophilin genes**	**8164**	**0.2923**	**-0.05734**	**0.01736**	**0.001336**	**-3.304**	**0.0009572**
6	C	rs2844479	31680935	HLA class III	8183	0.3890	-0.03031	0.01605	0.0004355	-1.888	0.05907
6	G	rs3130050	31726740	HLA class III	8178	0.1249	0.06717	0.02369	0.0009819	2.835	0.004598
6	T	rs185819	32158045	HLA class III	8178	0.4576	0.0516	0.01576	0.001309	3.274	0.001066
6	G	rs1776897	34302989	*HMGA1, LBH*	8183	0.0940	0.02524	0.02701	0.0001068	0.9348	0.3499
6	A	rs2814993	34726871	*C6orf106*	8091	0.1390	0.06941	0.02284	0.001141	3.039	0.002378
6	A	rs4713858	35510763	*ANKS1, TCP11, ZNF76, DEF6, SCUBE3*	8184	0.1730	-0.02058	0.02103	0.000117	-0.9786	0.3278
6	C	rs314263	105499438	*LIN28B, HACE1, BVES, POPDC3*	8184	0.3129	0.02821	0.01698	0.0003373	1.661	0.09665
**6**	**T**	**rs1490388**	**126877348**	***C6orf173/LOC387103***	**8179**	**0.4784**	**0.05315**	**0.01571**	**0.001398**	**3.383**	**0.0007196**
**6**	**G**	**rs3748069**	**142809326**	***GPR126***	**8184**	**0.3126**	**-0.06047**	**0.01695**	**0.001552**	**-3.566**	**0.0003641**
7	T	rs798544	2536343	*GNA12*	8184	0.2897	0.009501	0.01746	3.62E-05	0.5441	0.5864
7	C	rs1182188	2643226	*GNA12*	8184	0.2948	0.01528	0.01742	9.40E-05	0.8769	0.3806
7	A	rs849141	27958331	*JAZF1*	8180	0.2729	0.05199	0.01769	0.001055	2.939	0.0033
7	C	rs2282978	91909061	*CDK6, PEX1, GATAD1, ERVWE1*	8180	0.3562	-0.0002285	0.01639	2.38E-08	-0.01394	0.9889
7	C	rs11765954	91925346	*CDK6, PEX1, GATAD1, ERVWE1*	8183	0.2866	0.006002	0.01744	1.45E-05	0.3441	0.7307
8	C	rs10958476	57258362	*PLAG1, MOS, CHCHD7, RDHE2, RPS20, LYN, TGS1, PENK*	8158	0.2015	0.05689	0.01979	0.001012	2.874	0.004062
8	C	rs7846385	78322734	*PXMP3, ZFHX4*	8175	0.2753	0.004128	0.01761	6.72E-06	0.2344	0.8147
9	G	rs4448343	95345925	*PTCH1*	8182	0.3217	-0.007508	0.01686	2.42E-05	-0.4453	0.6561
9	A	rs4743034	106711908	*ZNF462*	8183	0.2250	0.01859	0.01872	0.0001206	0.9933	0.3206
12	C	rs8756	64646019	*HMGA2*	8175	0.4607	0.02308	0.01586	0.0002588	1.455	0.1458
12	G	rs3825199	92479422	*SOCS2, MRPL42, CRADD, UBE2N*	8183	0.2088	0.02109	0.0194	0.0001445	1.087	0.2769
13	C	rs1239947	50004556	*DLEU7*	8183	0.3287	0.03243	0.01678	0.0004563	1.933	0.05333
14	C	rs910316	74695795	*TMED10*	8184	0.4863	-0.02021	0.01577	0.0002006	-1.281	0.2002
14	C	rs7153027	91496975	*TRIP11, FBLN5, ATXN3, CPSF2*	8149	0.4316	-0.02966	0.0158	0.0004323	-1.877	0.06054
15	C	rs2554380	82106888	*ADAMTSL3, SH3GL3*	8067	0.1991	0.01455	0.02029	6.38E-05	0.7171	0.4734
15	T	rs11633371	87157836	*ACAN*	8184	0.4636	0.02862	0.01575	0.0004032	1.817	0.06929
15	A	rs4533267	98603794	*ADAMTS17*	8184	0.2933	-0.001093	0.01737	4.84E-07	-0.06294	0.9498
17	A	rs3760318	26271841	*CRLF3, ATAD5, CENTA2, RNF135*	8184	0.3821	-0.03009	0.01627	0.0004178	-1.849	0.06444
17	A	rs4794665	52205328	*NOG, DGKE, TRIM25, COIL, RISK*	8183	0.4756	0.006489	0.01569	2.09E-05	0.4135	0.6792
17	A	rs757608	56852059	*BCAS3, NACA2, TBX2, TBX4*	8126	0.3322	0.02008	0.01676	0.0001767	1.198	0.2309
18	G	rs4800148	18978326	*CABLES1, RBBP8, C18orf45*	8183	0.2043	-0.04987	0.01943	0.0008048	-2.567	0.01028
18	T	rs530550	45105636	*DYM*	8182	0.3572	-0.01124	0.01631	5.81E-05	-0.6892	0.4907
19	G	rs12459350	2127586	*DOT1L*	8179	0.4744	0.02751	0.01579	0.0003711	1.742	0.08149
20	A	rs967417	6568893	*BMP2*	8184	0.4495	-0.02479	0.01591	0.0002964	-1.558	0.1194
20	C	rs4911494	33435328	*UQCC, GDF5, CEP250, EIF6, MMP24*	8182	0.3864	0.05107	0.01621	0.001212	3.151	0.001633

The SNPs in bold are those that survived correction for multiple testing.

NMISS: number of individuals tested; MAF: minor allele frequency; BETA: regression coefficient for the test SNP; SE: standard error of the regression coefficient; R2: r^2 ^value in linear regression; T: test statistic; P: two-sided trend test *P*-value. The direction of effect is shown for the minor allele in each case.

In summary, sixteen of these SNPs yielded at least nominally significant association to height (*P *< 0.05), representing fifteen different loci with the same direction of effect as previously reported. Of these fifteen loci, variation at the *EFEMP1-PNPT1 *locus yielded the strongest association with *P *= 1.39×10^-5^, namely rs3791679.

With a slightly lower magnitude of association was *GPR126 *with rs3748069 yielding a *P *= 3.64×10^-4^, *C6orf173 *(also known as *LOC387103*) with rs1490388 yielding a *P *= 7.20×10^-4^, *SPAG17 *with 118574711 yielding a *P *= 7.27×10^-4 ^and the Histone class 1 gene cluster with rs10946808 yielding a *P *= 9.57×10^-4^.

Overall, in addition to these loci, we found evidence for association at the HLA class III region, *UQCC-GDF5, C6orf106*, *JAZF1, ZBTB38, PLAG1, C1orf19-GLT25D2, LCORL-NCAPG, CABLES1-RBBP8-C18orf45 *and *SCMH1 *loci. One could argue that we have carried out multiple testing in our height cohort for these previously reported SNPs, albeit at a number of magnitudes less than for a full GWA study. If we were to apply the strictest correction, i.e. the Bonferroni correction based on 51 SNPs, then *EFEMP1-PNPT1*, *GPR126*, *C6orf173*, *SPAG17 *and the Histone class 1 gene cluster would still be considered significant and their effects are consistent with the outcomes of the adult GWA studies.

It was also observed that SNPs residing at the 31 other loci did not reveal any evidence of association with height in our pediatric cohort, most notably *HMGA2*.

Finally, we investigated the sixteen significant SNPs further by testing for association between height Z-score and the genotype score, by summing the number of height increasing alleles across all these SNPs. The resulting *P*-value for the genotype score was < 2×10^-16 ^(Figure 1). The genotype score explains 1.64% of the total variation for height z-score. We also tested pair-wise interactions between the sixteen significant SNPs, but none of the interaction effects were significant, suggesting that these sixteen SNPs act additively on pediatric height.

## Discussion

We queried the existing dataset from our ongoing GWAS of pediatric height in European Americans for adult height loci uncovered in GWAS to date. We examined 51 single nucleotide polymorphisms (SNPs) corresponding to 46 genomic loci in 8,184 children with height measurements. Sixteen of these SNPs yielded at least nominally significant association to the trait, representing fifteen different loci.

One of the more notable results is the negative association with *HMGA2*. This gene is one of the most strongly associated loci with adult height[9] so its lack of association with childhood stature in this study is striking. We previously published a replication attempt with this locus and pediatric height when our cohort was substantially smaller[21]; at that time, we observed nominal association but it is clear that as our cohort has grown, this signal has failed to strengthen. Despite the wealth the evidence from adult GWA studies and from previous work with knock-out mouse models, it is of surprise not to observe association with *HMGA2*. However, when considering the age bins presented in Additional file 2: Supplemental Table S2, the T statistic generally increases with age, with the strongest value being for the 15-18 age group. Although none of these observations are significant, it may point to an age-specific effect at a particular point during childhood that is undetected in the overall analysis; however our large cohort size may still not be powered enough to tease out this effect.

For the loci we did not observe any evidence for association at all may be due to power issues, but could also indicate that they have a less pronounced role in a pediatric setting. In addition, only a portion of the published adult height loci have been independently and robustly replicated to date[22]. It should also be noted that childhood growth is an ongoing process where development factors may cloud detection at certain loci, including at the two rapid growth stages, where nutrition plays a major role in infant growth and hormone signaling impacts at puberty. Our study may lack power to detect stage specific association when using a mixed age childhood cohort; however we have presented the association results for specific age bins in Additional file 2: Supplemental Table S2.

From this analysis, it is clear that a number of loci previously reported from GWA analyses of adult height also play a role in our phenotype of interest. While these recently discovered loci unveil several new biomolecular pathways not previously associated with height, it is important to note that these well established genetic associations with stature explain very little of the genetic contribution for this pediatric phenotype, suggesting the existence of additional loci whose number and effect size remain unknown.

## Conclusions

Among 46 loci that have been reported to associate with adult height to date, at least 15 also contribute to the determination of height in childhood. Once our GWA study is complete, we will have the opportunity to look for other variants in the genome that are associated with height in childhood.

## Competing interests

The authors declare that they have no competing interests.

## Authors' contributions

JZ, HH and SFAG designed the study and supervised the data analysis and interpretation. JZ, ML, HZ and SFAG conducted the statistical analyses. CEK, CH, KAT, MLG, SD, ECF and FGO directed the genotyping and related sample handling. JPB, FDM, KW, PMS, JTG and BJG provided bioinformatics support. RMC and RIB coordinated the sample recruitment. JZ, ML, HH and SFAG drafted the manuscript. All the authors read and approved the final manuscript.

## Pre-publication history

The pre-publication history for this paper can be accessed here:

http://www.biomedcentral.com/1471-2350/11/96/prepub

## Supplementary Material

Additional File 1 **Supplemental Table S1: Surrogates used in this study **- as derived from the CEU HapMap. Click here for file

Additional File 2 **Supplemental Table S2: Quantitative association results for the candidate loci in the European American height cohort**. Data is presented separately for age bins defined for under 2s, 2-5, 6-10, 11-14 and 15-18 year olds, sorted by chromosomal location. Click here for file
