# Supplementary material for: The role of height-associated loci identified in genome wide association studies in the determination of pediatric stature
Source: BMC Med Genet. 2010 Jun 14;11:96. doi: 10.1186/1471-2350-11-96 (PMC2894790; doi:10.1186/1471-2350-11-96)
Supplement: Additional File 1 — Supplemental Table S1: Surrogates used in this study - as derived from the CEU HapMap. [file 1471-2350-11-96-S1.DOC]

**Supplemental Table S1. Surrogates used in this study** (derived from the CEU HapMap)

| **CHR** | **SNP on our Illumina Infinium™ II HumanHap550 BeadChip** | **SNP used in other system** | **r^2^** | **Genes Nearby** |
| --- | --- | --- | --- | --- |
| 1 | rs6663565 | rs6686842 | 1 | *SCMH1* |
| 1 | rs17038164 | rs12735613 | 0.92 | *SPAG17* |
| 1 | rs3942992 | rs1390401 | 1 | *ZNF678* |
| 3 | rs9841212 | rs10935120 | 1 | *ANAPC13, CEP63* |
| 6 | rs314263 | rs314268 | 1 | *LIN28B, HACE1, BVES, POPDC3* |
| 9 | rs4448343 | rs10512248 | 1 | *PTCH1* |
| 12 | rs8756 | rs1042725 | 0.873 | *HMGA2* |
| 15 | rs11633371 | rs8041863 | 1 | *ACAN* |
| 18 | rs530550 | rs8099594 | 0.87 | *DYM* |
| 19 | rs12459350 | rs12986413 | 1 | *DOT1L* |
| 20 | rs4911494 | rs6060369 | 0.964 | *UQCC, GDF5, CEP250, EIF6, MMP24* |
