# Supplementary material for: The role of height-associated loci identified in genome wide association studies in the determination of pediatric stature
Source: BMC Med Genet. 2010 Jun 14;11:96. doi: 10.1186/1471-2350-11-96 (PMC2894790; doi:10.1186/1471-2350-11-96)
Supplement: Additional File 2 — Supplemental Table S2: Quantitative association results for the candidate loci in the European American height cohort. Data is presented separately for age bins defined for under 2s, 2-5, 6-10, 11-14 and 15-18 year olds, sorted by chromosomal location. [file 1471-2350-11-96-S2.DOC]

**Supplemental Table S2a.** Quantitative association results for the candidate loci in the European American height cohort, under 2 years old (n=1,097), sorted by chromosomal location.

| **Chr** | **Minor**  **Allele** | **SNP** | **Position (Build 36)** | **Nearby genes(s)** | **NMISS** | **BETA** | **SE** | **R2** | **T** | **P** |
| --- | --- | --- | --- | --- | --- | --- | --- | --- | --- | --- |
| 1 | A | rs11809207 | 26205282 | *CATSPER4* | 1088 | 0.006082 | 0.05932 | 9.68E-06 | 0.1025 | 0.9184 |
| 1 | C | rs6663565 | 41232781 | *SCMH1* | 1097 | -0.001644 | 0.04386 | 1.28E-06 | -0.03749 | 0.9701 |
| 1 | C | rs17038164 | 118574711 | *SPAG17* | 1097 | -0.08065 | 0.04921 | 0.002447 | -1.639 | 0.1015 |
| 1 | G | rs11205277 | 146705945 | Histone class 2A, *MTMR11, SV2A, SF3B4* | 1097 | -0.01217 | 0.04316 | 7.26E-05 | -0.282 | 0.778 |
| 1 | G | rs678962 | 168921546 | *DNM3* | 1096 | -0.01774 | 0.05137 | 0.0001091 | -0.3454 | 0.7298 |
| 1 | A | rs2274432 | 180752602 | *C1orf19,GLT25D2* | 1077 | 0.001328 | 0.04726 | 7.35E-07 | 0.02811 | 0.9776 |
| 1 | A | rs3942992 | 224079131 | *ZNF678* | 1097 | -0.01795 | 0.05536 | 9.60E-05 | -0.3243 | 0.7458 |
| 2 | G | rs3791679 | 56008543 | *EFEMP1, PNPT1* | 1097 | -0.04029 | 0.04935 | 0.0006083 | -0.8164 | 0.4145 |
| 2 | T | rs1052483 | 219759853 | *IHH, CRYBA2, FEV, SLC23A3, TUBA1* | 1089 | -0.03259 | 0.07214 | 0.0001877 | -0.4518 | 0.6515 |
| 3 | C | rs9841212 | 135674636 | *ANAPC13, CEP63* | 1096 | 0.0787 | 0.04636 | 0.002627 | 1.698 | 0.08988 |
| 3 | A | rs6763931 | 142585531 | *ZBTB38* | 1097 | -0.01357 | 0.04377 | 8.78E-05 | -0.3101 | 0.7566 |
| 4 | T | rs6842303 | 17530324 | *LCORL, NCAPG* | 1097 | -0.02483 | 0.04982 | 0.0002269 | -0.4985 | 0.6182 |
| 4 | C | rs6830062 | 17693999 | *LCORL, NCAPG* | 1097 | 0.0227 | 0.05434 | 0.0001593 | 0.4177 | 0.6763 |
| 4 | A | rs1812175 | 145932449 | *HHIP* | 1095 | -0.02089 | 0.05954 | 0.0001126 | -0.3509 | 0.7257 |
| 5 | T | rs10472828 | 32924575 | *NPR3* | 1097 | -0.0232 | 0.04347 | 0.0002601 | -0.5337 | 0.5936 |
| 6 | A | rs12198986 | 7665058 | *BMP6* | 1097 | -0.02981 | 0.04381 | 0.0004227 | -0.6805 | 0.4963 |
| 6 | G | rs10946808 | 26341366 | Histone class 1, Butyrophilin genes | 1093 | -0.00338 | 0.04689 | 4.76E-06 | -0.07209 | 0.9425 |
| 6 | C | rs2844479 | 31680935 | HLA class III | 1097 | 0.02093 | 0.0429 | 0.0002173 | 0.4878 | 0.6258 |
| 6 | G | rs3130050 | 31726740 | HLA class III | 1097 | 0.008522 | 0.06273 | 1.69E-05 | 0.1358 | 0.892 |
| 6 | T | rs185819 | 32158045 | HLA class III | 1097 | 0.01051 | 0.04255 | 5.57E-05 | 0.247 | 0.8049 |
| 6 | G | rs1776897 | 34302989 | *HMGA1, LBH* | 1097 | -0.07629 | 0.07283 | 0.001001 | -1.048 | 0.2951 |
| 6 | A | rs2814993 | 34726871 | *C6orf106* | 1089 | 0.04849 | 0.06161 | 0.0005697 | 0.7871 | 0.4314 |
| 6 | A | rs4713858 | 35510763 | *ANKS1, TCP11, ZNF76, DEF6, SCUBE3* | 1097 | -0.03067 | 0.05622 | 0.0002716 | -0.5455 | 0.5855 |
| 6 | C | rs314263 | 105499438 | *LIN28B, HACE1, BVES, POPDC3* | 1097 | 0.02227 | 0.04407 | 0.0002331 | 0.5053 | 0.6134 |
| 6 | T | rs1490388 | 126877348 | *C6orf173 / LOC387103* | 1097 | 0.08239 | 0.0417 | 0.003553 | 1.976 | 0.04839 |
| 6 | G | rs3748069 | 142809326 | *GPR126* | 1097 | -0.01606 | 0.04626 | 0.00011 | -0.3471 | 0.7286 |
| 7 | T | rs798544 | 2536343 | *GNA12* | 1097 | -0.0929 | 0.04827 | 0.003371 | -1.924 | 0.05457 |
| 7 | C | rs1182188 | 2643226 | *GNA12* | 1097 | -0.07486 | 0.04793 | 0.002223 | -1.562 | 0.1186 |
| 7 | A | rs849141 | 27958331 | *JAZF1* | 1097 | -0.02837 | 0.04772 | 0.0003226 | -0.5944 | 0.5524 |
| 7 | C | rs2282978 | 91909061 | *CDK6, PEX1, GATAD1, ERVWE1* | 1097 | 0.01301 | 0.04387 | 8.03E-05 | 0.2965 | 0.7669 |
| 7 | C | rs11765954 | 91925346 | *CDK6, PEX1, GATAD1, ERVWE1* | 1097 | 0.02624 | 0.04617 | 0.0002948 | 0.5682 | 0.57 |
| 8 | C | rs10958476 | 57258362 | *PLAG1, MOS, CHCHD7, RDHE2, RPS20, LYN, TGS1, PENK* | 1092 | 0.01413 | 0.05467 | 6.13E-05 | 0.2584 | 0.7961 |
| 8 | C | rs7846385 | 78322734 | *PXMP3, ZFHX4* | 1095 | -0.0824 | 0.0491 | 0.00257 | -1.678 | 0.09363 |
| 9 | G | rs4448343 | 95345925 | *PTCH1* | 1097 | -0.04132 | 0.0464 | 0.0007237 | -0.8905 | 0.3734 |
| 9 | A | rs4743034 | 106711908 | *ZNF462* | 1097 | -0.05991 | 0.05151 | 0.001234 | -1.163 | 0.245 |
| 12 | C | rs8756 | 64646019 | *HMGA2* | 1094 | -0.04316 | 0.04283 | 0.0009289 | -1.008 | 0.3139 |
| 12 | G | rs3825199 | 92479422 | *SOCS2, MRPL42, CRADD, UBE2N* | 1097 | -0.03735 | 0.05322 | 0.0004497 | -0.7019 | 0.4829 |
| 13 | C | rs1239947 | 50004556 | *DLEU7* | 1097 | -0.03174 | 0.04548 | 0.0004445 | -0.6978 | 0.4854 |
| 14 | C | rs910316 | 74695795 | *TMED10* | 1097 | -0.02716 | 0.04278 | 0.0003679 | -0.6348 | 0.5257 |
| 14 | C | rs7153027 | 91496975 | *TRIP11, FBLN5, ATXN3, CPSF2* | 1095 | 0.0002273 | 0.04245 | 2.62E-08 | 0.005354 | 0.9957 |
| 15 | C | rs2554380 | 82106888 | *ADAMTSL3, SH3GL3* | 1083 | 0.05916 | 0.05601 | 0.001031 | 1.056 | 0.2911 |
| 15 | T | rs11633371 | 87157836 | *ACAN* | 1097 | 0.01101 | 0.04297 | 6.00E-05 | 0.2562 | 0.7978 |
| 15 | A | rs4533267 | 98603794 | *ADAMTS17* | 1097 | 0.03653 | 0.04739 | 0.0005424 | 0.7709 | 0.441 |
| 17 | A | rs3760318 | 26271841 | *CRLF3, ATAD5, CENTA2, RNF135* | 1097 | -0.04181 | 0.0448 | 0.0007946 | -0.9331 | 0.351 |
| 17 | A | rs4794665 | 52205328 | *NOG, DGKE, TRIM25, COIL, RISK* | 1096 | 0.0787 | 0.04226 | 0.003161 | 1.862 | 0.06281 |
| 17 | A | rs757608 | 56852059 | *BCAS3, NACA2, TBX2, TBX4* | 1093 | 0.006058 | 0.04517 | 1.65E-05 | 0.1341 | 0.8933 |
| 18 | G | rs4800148 | 18978326 | *CABLES1, RBBP8, C18orf45* | 1097 | -0.1051 | 0.05344 | 0.003516 | -1.966 | 0.04959 |
| 18 | T | rs530550 | 45105636 | *DYM* | 1097 | -0.02374 | 0.04576 | 0.0002458 | -0.5189 | 0.604 |
| 19 | G | rs12459350 | 2127586 | *DOT1L* | 1097 | -0.03001 | 0.04381 | 0.0004283 | -0.685 | 0.4935 |
| 20 | A | rs967417 | 6568893 | *BMP2* | 1097 | 0.00358 | 0.04399 | 6.05E-06 | 0.08138 | 0.9352 |
| 20 | C | rs4911494 | 33435328 | *UQCC, GDF5, CEP250, EIF6, MMP24* | 1097 | 0.07649 | 0.04522 | 0.002606 | 1.692 | 0.091 |

NMISS: number of individuals tested; BETA: regression coefficient for the test SNP; SE: standard error of the regression coefficient; R2: r^2^ value in linear regression; T: test statistic; P: two-sided trend test *P*-value. The direction of effect is shown for the minor allele in each case.

**Supplemental Table S2b.** Quantitative association results for the candidate loci in the European American height cohort, ages 2-5 years old (n=1,933), sorted by chromosomal location.

| **Chr** | **Minor**  **Allele** | **SNP** | **Position (Build 36)** | **Nearby genes(s)** | **NMISS** | **BETA** | **SE** | **R2** | **T** | **P** |
| --- | --- | --- | --- | --- | --- | --- | --- | --- | --- | --- |
| 1 | A | rs11809207 | 26205282 | *CATSPER4* | 1919 | 0.09697 | 0.04232 | 0.002732 | 2.292 | 0.02203 |
| 1 | C | rs6663565 | 41232781 | *SCMH1* | 1933 | 0.01161 | 0.03266 | 6.54E-05 | 0.3555 | 0.7223 |
| 1 | C | rs17038164 | 118574711 | *SPAG17* | 1932 | -0.0939 | 0.03585 | 0.003542 | -2.619 | 0.008884 |
| 1 | G | rs11205277 | 146705945 | Histone class 2A, *MTMR11, SV2A, SF3B4* | 1931 | 0.008356 | 0.03293 | 3.34E-05 | 0.2538 | 0.7997 |
| 1 | G | rs678962 | 168921546 | *DNM3* | 1932 | 0.009332 | 0.03936 | 2.91E-05 | 0.2371 | 0.8126 |
| 1 | A | rs2274432 | 180752602 | *C1orf19,GLT25D2* | 1895 | 0.02248 | 0.03472 | 0.0002214 | 0.6474 | 0.5175 |
| 1 | A | rs3942992 | 224079131 | *ZNF678* | 1932 | -0.006669 | 0.04348 | 1.22E-05 | -0.1534 | 0.8781 |
| 2 | G | rs3791679 | 56008543 | *EFEMP1, PNPT1* | 1933 | 0.006914 | 0.03669 | 1.84E-05 | 0.1885 | 0.8505 |
| 2 | T | rs1052483 | 219759853 | *IHH, CRYBA2, FEV, SLC23A3, TUBA1* | 1918 | -0.0299 | 0.05818 | 0.0001379 | -0.514 | 0.6073 |
| 3 | C | rs9841212 | 135674636 | *ANAPC13, CEP63* | 1929 | 0.03911 | 0.0343 | 0.0006742 | 1.14 | 0.2544 |
| 3 | A | rs6763931 | 142585531 | *ZBTB38* | 1929 | 0.06186 | 0.03243 | 0.001885 | 1.908 | 0.05659 |
| 4 | T | rs6842303 | 17530324 | *LCORL, NCAPG* | 1929 | -0.007186 | 0.03717 | 1.94E-05 | -0.1933 | 0.8467 |
| 4 | C | rs6830062 | 17693999 | *LCORL, NCAPG* | 1933 | -0.0767 | 0.04045 | 0.001859 | -1.896 | 0.05807 |
| 4 | A | rs1812175 | 145932449 | *HHIP* | 1928 | -0.03155 | 0.04378 | 0.0002695 | -0.7205 | 0.4713 |
| 5 | T | rs10472828 | 32924575 | *NPR3* | 1931 | -0.01628 | 0.03291 | 0.0001269 | -0.4947 | 0.6208 |
| 6 | A | rs12198986 | 7665058 | *BMP6* | 1933 | 0.01389 | 0.03249 | 9.46E-05 | 0.4273 | 0.6692 |
| 6 | G | rs10946808 | 26341366 | Histone class 1, Butyrophilin genes | 1929 | -0.04004 | 0.03634 | 0.0006295 | -1.102 | 0.2707 |
| 6 | C | rs2844479 | 31680935 | HLA class III | 1932 | -0.0614 | 0.03282 | 0.00181 | -1.871 | 0.06154 |
| 6 | G | rs3130050 | 31726740 | HLA class III | 1931 | -0.004007 | 0.04911 | 3.45E-06 | -0.0816 | 0.935 |
| 6 | T | rs185819 | 32158045 | HLA class III | 1933 | 0.056 | 0.03271 | 0.001516 | 1.712 | 0.08705 |
| 6 | G | rs1776897 | 34302989 | *HMGA1, LBH* | 1933 | -0.02742 | 0.05675 | 0.0001209 | -0.4832 | 0.629 |
| 6 | A | rs2814993 | 34726871 | *C6orf106* | 1912 | 0.08407 | 0.04572 | 0.001767 | 1.839 | 0.0661 |
| 6 | A | rs4713858 | 35510763 | *ANKS1, TCP11, ZNF76, DEF6, SCUBE3* | 1933 | -0.05553 | 0.0443 | 0.0008133 | -1.254 | 0.2101 |
| 6 | C | rs314263 | 105499438 | *LIN28B, HACE1, BVES, POPDC3* | 1933 | 0.01572 | 0.03474 | 0.000106 | 0.4525 | 0.6509 |
| 6 | T | rs1490388 | 126877348 | *C6orf173 / LOC387103* | 1932 | 0.0713 | 0.03293 | 0.002423 | 2.165 | 0.03049 |
| 6 | G | rs3748069 | 142809326 | *GPR126* | 1933 | -0.06528 | 0.03572 | 0.001727 | -1.828 | 0.06777 |
| 7 | T | rs798544 | 2536343 | *GNA12* | 1933 | 0.0467 | 0.03572 | 0.0008843 | 1.307 | 0.1913 |
| 7 | C | rs1182188 | 2643226 | *GNA12* | 1933 | 0.04944 | 0.03591 | 0.0009805 | 1.377 | 0.1688 |
| 7 | A | rs849141 | 27958331 | *JAZF1* | 1931 | 0.0983 | 0.03681 | 0.003683 | 2.67 | 0.007639 |
| 7 | C | rs2282978 | 91909061 | *CDK6, PEX1, GATAD1, ERVWE1* | 1931 | -0.009406 | 0.03366 | 4.05E-05 | -0.2795 | 0.7799 |
| 7 | C | rs11765954 | 91925346 | *CDK6, PEX1, GATAD1, ERVWE1* | 1933 | 0.01297 | 0.03583 | 6.78E-05 | 0.3619 | 0.7174 |
| 8 | C | rs10958476 | 57258362 | *PLAG1, MOS, CHCHD7, RDHE2, RPS20, LYN, TGS1, PENK* | 1929 | 0.05446 | 0.04062 | 0.000932 | 1.341 | 0.1801 |
| 8 | C | rs7846385 | 78322734 | *PXMP3, ZFHX4* | 1930 | 0.007 | 0.03609 | 1.95E-05 | 0.1939 | 0.8462 |
| 9 | G | rs4448343 | 95345925 | *PTCH1* | 1932 | 0.04878 | 0.03457 | 0.00103 | 1.411 | 0.1584 |
| 9 | A | rs4743034 | 106711908 | *ZNF462* | 1933 | 0.05936 | 0.03869 | 0.001217 | 1.534 | 0.1252 |
| 12 | C | rs8756 | 64646019 | *HMGA2* | 1929 | 0.02368 | 0.03292 | 0.0002684 | 0.7193 | 0.472 |
| 12 | G | rs3825199 | 92479422 | *SOCS2, MRPL42, CRADD, UBE2N* | 1932 | -0.02965 | 0.03963 | 0.0002899 | -0.7481 | 0.4545 |
| 13 | C | rs1239947 | 50004556 | *DLEU7* | 1933 | 0.07294 | 0.03465 | 0.00229 | 2.105 | 0.03539 |
| 14 | C | rs910316 | 74695795 | *TMED10* | 1933 | -0.004533 | 0.03262 | 1.00E-05 | -0.139 | 0.8895 |
| 14 | C | rs7153027 | 91496975 | *TRIP11, FBLN5, ATXN3, CPSF2* | 1923 | -0.009875 | 0.03249 | 4.81E-05 | -0.3039 | 0.7612 |
| 15 | C | rs2554380 | 82106888 | *ADAMTSL3, SH3GL3* | 1910 | -0.05359 | 0.04269 | 0.0008254 | -1.255 | 0.2095 |
| 15 | T | rs11633371 | 87157836 | *ACAN* | 1933 | 0.0297 | 0.03293 | 0.0004208 | 0.9016 | 0.3674 |
| 15 | A | rs4533267 | 98603794 | *ADAMTS17* | 1933 | -0.04501 | 0.03615 | 0.0008023 | -1.245 | 0.2132 |
| 17 | A | rs3760318 | 26271841 | *CRLF3, ATAD5, CENTA2, RNF135* | 1933 | -0.07138 | 0.03414 | 0.002259 | -2.091 | 0.03667 |
| 17 | A | rs4794665 | 52205328 | *NOG, DGKE, TRIM25, COIL, RISK* | 1933 | -0.03902 | 0.03232 | 0.0007541 | -1.207 | 0.2275 |
| 17 | A | rs757608 | 56852059 | *BCAS3, NACA2, TBX2, TBX4* | 1919 | -0.0078 | 0.03485 | 2.61E-05 | -0.2238 | 0.8229 |
| 18 | G | rs4800148 | 18978326 | *CABLES1, RBBP8, C18orf45* | 1933 | -0.008458 | 0.03989 | 2.33E-05 | -0.212 | 0.8321 |
| 18 | T | rs530550 | 45105636 | *DYM* | 1932 | 0.04477 | 0.03348 | 0.0009253 | 1.337 | 0.1814 |
| 19 | G | rs12459350 | 2127586 | *DOT1L* | 1933 | 0.05941 | 0.03246 | 0.001732 | 1.83 | 0.06736 |
| 20 | A | rs967417 | 6568893 | *BMP2* | 1933 | -0.03658 | 0.03272 | 0.0006469 | -1.118 | 0.2637 |
| 20 | C | rs4911494 | 33435328 | *UQCC, GDF5, CEP250, EIF6, MMP24* | 1932 | -0.01941 | 0.03348 | 0.000174 | -0.5796 | 0.5622 |

NMISS: number of individuals tested; BETA: regression coefficient for the test SNP; SE: standard error of the regression coefficient; R2: r^2^ value in linear regression; T: test statistic; P: two-sided trend test *P*-value. The direction of effect is shown for the minor allele in each case.

**Supplemental Table S2c.** Quantitative association results for the candidate loci in the European American height cohort, ages 6-10 years old (n=1,829), sorted by chromosomal location.

| **Chr** | **Minor**  **Allele** | **SNP** | **Position (Build 36)** | **Nearby genes(s)** | **NMISS** | **BETA** | **SE** | **R2** | **T** | **P** |
| --- | --- | --- | --- | --- | --- | --- | --- | --- | --- | --- |
| 1 | A | rs11809207 | 26205282 | *CATSPER4* | 1812 | -0.02669 | 0.04496 | 0.0001946 | -0.5936 | 0.5529 |
| 1 | C | rs6663565 | 41232781 | *SCMH1* | 1829 | 0.08526 | 0.03365 | 0.003501 | 2.534 | 0.01137 |
| 1 | C | rs17038164 | 118574711 | *SPAG17* | 1829 | -0.007229 | 0.03833 | 1.95E-05 | -0.1886 | 0.8504 |
| 1 | G | rs11205277 | 146705945 | Histone class 2A, *MTMR11, SV2A, SF3B4* | 1829 | 0.01413 | 0.03307 | 9.99E-05 | 0.4272 | 0.6693 |
| 1 | G | rs678962 | 168921546 | *DNM3* | 1829 | -0.02243 | 0.04052 | 0.0001677 | -0.5535 | 0.58 |
| 1 | A | rs2274432 | 180752602 | *C1orf19,GLT25D2* | 1771 | 0.07416 | 0.03681 | 0.002289 | 2.015 | 0.0441 |
| 1 | A | rs3942992 | 224079131 | *ZNF678* | 1827 | 0.01615 | 0.04315 | 7.67E-05 | 0.3742 | 0.7083 |
| 2 | G | rs3791679 | 56008543 | *EFEMP1, PNPT1* | 1829 | -0.06476 | 0.03835 | 0.001558 | -1.689 | 0.09145 |
| 2 | T | rs1052483 | 219759853 | *IHH, CRYBA2, FEV, SLC23A3, TUBA1* | 1813 | -0.02903 | 0.0571 | 0.0001428 | -0.5085 | 0.6112 |
| 3 | C | rs9841212 | 135674636 | *ANAPC13, CEP63* | 1821 | 0.008817 | 0.03478 | 3.53E-05 | 0.2535 | 0.7999 |
| 3 | A | rs6763931 | 142585531 | *ZBTB38* | 1827 | 0.03672 | 0.03325 | 0.0006679 | 1.104 | 0.2695 |
| 4 | T | rs6842303 | 17530324 | *LCORL, NCAPG* | 1825 | 0.06197 | 0.03906 | 0.001379 | 1.586 | 0.1128 |
| 4 | C | rs6830062 | 17693999 | *LCORL, NCAPG* | 1829 | -0.09024 | 0.0428 | 0.002427 | -2.108 | 0.03512 |
| 4 | A | rs1812175 | 145932449 | *HHIP* | 1829 | 0.01348 | 0.0452 | 4.87E-05 | 0.2982 | 0.7656 |
| 5 | T | rs10472828 | 32924575 | *NPR3* | 1829 | 0.01791 | 0.03363 | 0.0001553 | 0.5328 | 0.5943 |
| 6 | A | rs12198986 | 7665058 | *BMP6* | 1829 | -0.0009175 | 0.03383 | 4.03E-07 | -0.02712 | 0.9784 |
| 6 | G | rs10946808 | 26341366 | Histone class 1, Butyrophilin genes | 1822 | -0.08823 | 0.03689 | 0.003133 | -2.392 | 0.01687 |
| 6 | C | rs2844479 | 31680935 | HLA class III | 1829 | -0.04112 | 0.03459 | 0.0007731 | -1.189 | 0.2346 |
| 6 | G | rs3130050 | 31726740 | HLA class III | 1828 | 0.04924 | 0.05046 | 0.0005213 | 0.9759 | 0.3292 |
| 6 | T | rs185819 | 32158045 | HLA class III | 1828 | 0.004355 | 0.03396 | 9.01E-06 | 0.1282 | 0.898 |
| 6 | G | rs1776897 | 34302989 | *HMGA1, LBH* | 1829 | 0.06286 | 0.05662 | 0.0006741 | 1.11 | 0.2671 |
| 6 | A | rs2814993 | 34726871 | *C6orf106* | 1808 | 0.0004537 | 0.04787 | 4.98E-08 | 0.009479 | 0.9924 |
| 6 | A | rs4713858 | 35510763 | *ANKS1, TCP11, ZNF76, DEF6, SCUBE3* | 1829 | 0.04004 | 0.04352 | 0.0004632 | 0.9201 | 0.3576 |
| 6 | C | rs314263 | 105499438 | *LIN28B, HACE1, BVES, POPDC3* | 1829 | 0.05179 | 0.03671 | 0.001088 | 1.411 | 0.1585 |
| 6 | T | rs1490388 | 126877348 | *C6orf173 / LOC387103* | 1828 | 0.03905 | 0.03352 | 0.0007429 | 1.165 | 0.2441 |
| 6 | G | rs3748069 | 142809326 | *GPR126* | 1829 | -0.06986 | 0.03554 | 0.00211 | -1.965 | 0.04951 |
| 7 | T | rs798544 | 2536343 | *GNA12* | 1829 | 0.04024 | 0.03655 | 0.0006627 | 1.101 | 0.2712 |
| 7 | C | rs1182188 | 2643226 | *GNA12* | 1829 | 0.0477 | 0.03618 | 0.0009504 | 1.318 | 0.1876 |
| 7 | A | rs849141 | 27958331 | *JAZF1* | 1827 | 0.05443 | 0.03722 | 0.00117 | 1.462 | 0.1438 |
| 7 | C | rs2282978 | 91909061 | *CDK6, PEX1, GATAD1, ERVWE1* | 1828 | -0.003773 | 0.03527 | 6.27E-06 | -0.107 | 0.9148 |
| 7 | C | rs11765954 | 91925346 | *CDK6, PEX1, GATAD1, ERVWE1* | 1828 | -0.007184 | 0.0376 | 2.00E-05 | -0.1911 | 0.8485 |
| 8 | C | rs10958476 | 57258362 | *PLAG1, MOS, CHCHD7, RDHE2, RPS20, LYN, TGS1, PENK* | 1823 | -0.01218 | 0.04139 | 4.76E-05 | -0.2944 | 0.7685 |
| 8 | C | rs7846385 | 78322734 | *PXMP3, ZFHX4* | 1828 | 0.04125 | 0.03694 | 0.0006824 | 1.117 | 0.2643 |
| 9 | G | rs4448343 | 95345925 | *PTCH1* | 1829 | -0.05825 | 0.03599 | 0.001432 | -1.619 | 0.1057 |
| 9 | A | rs4743034 | 106711908 | *ZNF462* | 1828 | 0.0191 | 0.03903 | 0.0001311 | 0.4893 | 0.6247 |
| 12 | C | rs8756 | 64646019 | *HMGA2* | 1827 | 0.03854 | 0.03402 | 0.0007027 | 1.133 | 0.2574 |
| 12 | G | rs3825199 | 92479422 | *SOCS2, MRPL42, CRADD, UBE2N* | 1829 | 0.05256 | 0.04051 | 0.0009207 | 1.298 | 0.1946 |
| 13 | C | rs1239947 | 50004556 | *DLEU7* | 1829 | -0.003662 | 0.03534 | 5.88E-06 | -0.1036 | 0.9175 |
| 14 | C | rs910316 | 74695795 | *TMED10* | 1829 | -0.04991 | 0.03315 | 0.001239 | -1.506 | 0.1323 |
| 14 | C | rs7153027 | 91496975 | *TRIP11, FBLN5, ATXN3, CPSF2* | 1819 | -0.05589 | 0.03387 | 0.001496 | -1.65 | 0.09913 |
| 15 | C | rs2554380 | 82106888 | *ADAMTSL3, SH3GL3* | 1792 | -0.015 | 0.0424 | 6.99E-05 | -0.3538 | 0.7235 |
| 15 | T | rs11633371 | 87157836 | *ACAN* | 1829 | 0.0003531 | 0.03299 | 6.27E-08 | 0.01071 | 0.9915 |
| 15 | A | rs4533267 | 98603794 | *ADAMTS17* | 1829 | -0.02287 | 0.03621 | 0.0002184 | -0.6317 | 0.5277 |
| 17 | A | rs3760318 | 26271841 | *CRLF3, ATAD5, CENTA2, RNF135* | 1829 | 0.03842 | 0.03469 | 0.0006709 | 1.107 | 0.2682 |
| 17 | A | rs4794665 | 52205328 | *NOG, DGKE, TRIM25, COIL, RISK* | 1829 | 0.01741 | 0.03329 | 0.0001496 | 0.5229 | 0.6011 |
| 17 | A | rs757608 | 56852059 | *BCAS3, NACA2, TBX2, TBX4* | 1816 | 0.02538 | 0.03539 | 0.0002834 | 0.7171 | 0.4734 |
| 18 | G | rs4800148 | 18978326 | *CABLES1, RBBP8, C18orf45* | 1829 | -0.01962 | 0.04065 | 0.0001276 | -0.4828 | 0.6293 |
| 18 | T | rs530550 | 45105636 | *DYM* | 1829 | -0.002769 | 0.03478 | 3.47E-06 | -0.07964 | 0.9365 |
| 19 | G | rs12459350 | 2127586 | *DOT1L* | 1827 | 0.0303 | 0.03319 | 0.0004564 | 0.9129 | 0.3614 |
| 20 | A | rs967417 | 6568893 | *BMP2* | 1829 | -0.08004 | 0.03395 | 0.003032 | -2.357 | 0.01852 |
| 20 | C | rs4911494 | 33435328 | *UQCC, GDF5, CEP250, EIF6, MMP24* | 1829 | 0.07222 | 0.03379 | 0.002494 | 2.137 | 0.03271 |

NMISS: number of individuals tested; BETA: regression coefficient for the test SNP; SE: standard error of the regression coefficient; R2: r^2^ value in linear regression; T: test statistic; P: two-sided trend test *P*-value. The direction of effect is shown for the minor allele in each case.

**Supplemental Table S2d.** Quantitative association results for the candidate loci in the European American height cohort, ages 11-14 years old (n=1,726), sorted by chromosomal location.

| **Chr** | **Minor**  **Allele** | **SNP** | **Position (Build 36)** | **Nearby genes(s)** | **NMISS** | **BETA** | **SE** | **R2** | **T** | **P** |
| --- | --- | --- | --- | --- | --- | --- | --- | --- | --- | --- |
| 1 | A | rs11809207 | 26205282 | *CATSPER4* | 1712 | 0.02274 | 0.04714 | 0.0001361 | 0.4824 | 0.6296 |
| 1 | C | rs6663565 | 41232781 | *SCMH1* | 1726 | 0.02406 | 0.03431 | 0.0002851 | 0.7012 | 0.4833 |
| 1 | C | rs17038164 | 118574711 | *SPAG17* | 1725 | -0.06599 | 0.03928 | 0.001635 | -1.68 | 0.09313 |
| 1 | G | rs11205277 | 146705945 | Histone class 2A, *MTMR11, SV2A, SF3B4* | 1726 | -0.01478 | 0.03433 | 0.0001075 | -0.4305 | 0.6669 |
| 1 | G | rs678962 | 168921546 | *DNM3* | 1725 | 0.111 | 0.04143 | 0.004149 | 2.679 | 0.007446 |
| 1 | A | rs2274432 | 180752602 | *C1orf19,GLT25D2* | 1668 | 0.06559 | 0.03758 | 0.001825 | 1.745 | 0.08112 |
| 1 | A | rs3942992 | 224079131 | *ZNF678* | 1726 | 0.01174 | 0.0456 | 3.84E-05 | 0.2574 | 0.7969 |
| 2 | G | rs3791679 | 56008543 | *EFEMP1, PNPT1* | 1724 | -0.1312 | 0.03897 | 0.006536 | -3.366 | 0.0007795 |
| 2 | T | rs1052483 | 219759853 | *IHH, CRYBA2, FEV, SLC23A3, TUBA1* | 1707 | -0.06917 | 0.05524 | 0.0009188 | -1.252 | 0.2107 |
| 3 | C | rs9841212 | 135674636 | *ANAPC13, CEP63* | 1713 | -0.07843 | 0.03729 | 0.002579 | -2.103 | 0.03558 |
| 3 | A | rs6763931 | 142585531 | *ZBTB38* | 1725 | 0.06588 | 0.03507 | 0.002044 | 1.879 | 0.06048 |
| 4 | T | rs6842303 | 17530324 | *LCORL, NCAPG* | 1725 | 0.03534 | 0.03983 | 0.0004568 | 0.8874 | 0.375 |
| 4 | C | rs6830062 | 17693999 | *LCORL, NCAPG* | 1726 | -0.08807 | 0.04426 | 0.002291 | -1.99 | 0.04676 |
| 4 | A | rs1812175 | 145932449 | *HHIP* | 1723 | -0.03599 | 0.04643 | 0.000349 | -0.7751 | 0.4384 |
| 5 | T | rs10472828 | 32924575 | *NPR3* | 1726 | -0.01828 | 0.03441 | 0.0001637 | -0.5312 | 0.5953 |
| 6 | A | rs12198986 | 7665058 | *BMP6* | 1726 | -0.01821 | 0.03551 | 0.0001525 | -0.5127 | 0.6082 |
| 6 | G | rs10946808 | 26341366 | Histone class 1, Butyrophilin genes | 1725 | -0.05496 | 0.03788 | 0.001221 | -1.451 | 0.1469 |
| 6 | C | rs2844479 | 31680935 | HLA class III | 1726 | -0.00257 | 0.03543 | 3.05E-06 | -0.07254 | 0.9422 |
| 6 | G | rs3130050 | 31726740 | HLA class III | 1725 | 0.1401 | 0.05245 | 0.004122 | 2.671 | 0.007644 |
| 6 | T | rs185819 | 32158045 | HLA class III | 1724 | 0.04875 | 0.03414 | 0.001182 | 1.428 | 0.1535 |
| 6 | G | rs1776897 | 34302989 | *HMGA1, LBH* | 1726 | 0.08496 | 0.05934 | 0.001188 | 1.432 | 0.1524 |
| 6 | A | rs2814993 | 34726871 | *C6orf106* | 1706 | 0.07884 | 0.05045 | 0.001431 | 1.563 | 0.1183 |
| 6 | A | rs4713858 | 35510763 | *ANKS1, TCP11, ZNF76, DEF6, SCUBE3* | 1726 | 0.03197 | 0.0455 | 0.0002862 | 0.7025 | 0.4824 |
| 6 | C | rs314263 | 105499438 | *LIN28B, HACE1, BVES, POPDC3* | 1726 | 0.03036 | 0.03722 | 0.000386 | 0.8159 | 0.4147 |
| 6 | T | rs1490388 | 126877348 | *C6orf173 / LOC387103* | 1726 | 0.04923 | 0.0343 | 0.001194 | 1.435 | 0.1514 |
| 6 | G | rs3748069 | 142809326 | *GPR126* | 1726 | -0.05765 | 0.03734 | 0.001381 | -1.544 | 0.1228 |
| 7 | T | rs798544 | 2536343 | *GNA12* | 1726 | 0.002269 | 0.03815 | 2.05E-06 | 0.05948 | 0.9526 |
| 7 | C | rs1182188 | 2643226 | *GNA12* | 1726 | 0.007334 | 0.03799 | 2.16E-05 | 0.1931 | 0.8469 |
| 7 | A | rs849141 | 27958331 | *JAZF1* | 1726 | 0.07199 | 0.03886 | 0.001987 | 1.853 | 0.06408 |
| 7 | C | rs2282978 | 91909061 | *CDK6, PEX1, GATAD1, ERVWE1* | 1725 | -0.005576 | 0.0358 | 1.41E-05 | -0.1557 | 0.8763 |
| 7 | C | rs11765954 | 91925346 | *CDK6, PEX1, GATAD1, ERVWE1* | 1726 | -0.01169 | 0.03851 | 5.34E-05 | -0.3035 | 0.7616 |
| 8 | C | rs10958476 | 57258362 | *PLAG1, MOS, CHCHD7, RDHE2, RPS20, LYN, TGS1, PENK* | 1720 | 0.08749 | 0.04392 | 0.002304 | 1.992 | 0.04655 |
| 8 | C | rs7846385 | 78322734 | *PXMP3, ZFHX4* | 1723 | 0.04856 | 0.0387 | 0.0009139 | 1.255 | 0.2098 |
| 9 | G | rs4448343 | 95345925 | *PTCH1* | 1725 | -0.05133 | 0.03711 | 0.001109 | -1.383 | 0.1668 |
| 9 | A | rs4743034 | 106711908 | *ZNF462* | 1726 | 0.01334 | 0.04079 | 6.20E-05 | 0.327 | 0.7437 |
| 12 | C | rs8756 | 64646019 | *HMGA2* | 1726 | 0.02275 | 0.03482 | 0.0002475 | 0.6533 | 0.5137 |
| 12 | G | rs3825199 | 92479422 | *SOCS2, MRPL42, CRADD, UBE2N* | 1726 | 0.02314 | 0.04262 | 0.000171 | 0.543 | 0.5872 |
| 13 | C | rs1239947 | 50004556 | *DLEU7* | 1726 | 0.02943 | 0.03678 | 0.0003712 | 0.8002 | 0.4237 |
| 14 | C | rs910316 | 74695795 | *TMED10* | 1726 | -0.01563 | 0.0352 | 0.0001144 | -0.4441 | 0.657 |
| 14 | C | rs7153027 | 91496975 | *TRIP11, FBLN5, ATXN3, CPSF2* | 1720 | -0.04688 | 0.03431 | 0.001085 | -1.366 | 0.1721 |
| 15 | C | rs2554380 | 82106888 | *ADAMTSL3, SH3GL3* | 1703 | 0.0837 | 0.04299 | 0.002224 | 1.947 | 0.0517 |
| 15 | T | rs11633371 | 87157836 | *ACAN* | 1726 | 0.02933 | 0.03436 | 0.0004224 | 0.8535 | 0.3935 |
| 15 | A | rs4533267 | 98603794 | *ADAMTS17* | 1726 | 0.01993 | 0.03834 | 0.0001567 | 0.5198 | 0.6033 |
| 17 | A | rs3760318 | 26271841 | *CRLF3, ATAD5, CENTA2, RNF135* | 1726 | -0.03493 | 0.03516 | 0.0005722 | -0.9935 | 0.3206 |
| 17 | A | rs4794665 | 52205328 | *NOG, DGKE, TRIM25, COIL, RISK* | 1726 | 0.01032 | 0.03477 | 5.11E-05 | 0.2969 | 0.7665 |
| 17 | A | rs757608 | 56852059 | *BCAS3, NACA2, TBX2, TBX4* | 1713 | 0.04063 | 0.03637 | 0.0007287 | 1.117 | 0.2641 |
| 18 | G | rs4800148 | 18978326 | *CABLES1, RBBP8, C18orf45* | 1725 | -0.0788 | 0.04331 | 0.001918 | -1.82 | 0.069 |
| 18 | T | rs530550 | 45105636 | *DYM* | 1725 | -0.03732 | 0.03548 | 0.0006416 | -1.052 | 0.2931 |
| 19 | G | rs12459350 | 2127586 | *DOT1L* | 1725 | 0.04883 | 0.03441 | 0.001167 | 1.419 | 0.1561 |
| 20 | A | rs967417 | 6568893 | *BMP2* | 1726 | -0.01706 | 0.03453 | 0.0001415 | -0.4939 | 0.6215 |
| 20 | C | rs4911494 | 33435328 | *UQCC, GDF5, CEP250, EIF6, MMP24* | 1726 | 0.04467 | 0.03465 | 0.000963 | 1.289 | 0.1975 |

NMISS: number of individuals tested; BETA: regression coefficient for the test SNP; SE: standard error of the regression coefficient; R2: r^2^ value in linear regression; T: test statistic; P: two-sided trend test *P*-value. The direction of effect is shown for the minor allele in each case.

**Supplemental Table S2e.** Quantitative association results for the candidate loci in the European American height cohort, ages 15-18 years old (n=1,599), sorted by chromosomal location.

| **Chr** | **Minor**  **Allele** | **SNP** | **Position (Build 36)** | **Nearby genes(s)** | **NMISS** | **BETA** | **SE** | **R2** | **T** | **P** |
| --- | --- | --- | --- | --- | --- | --- | --- | --- | --- | --- |
| 1 | A | rs11809207 | 26205282 | *CATSPER4* | 1575 | 0.01894 | 0.04911 | 9.45E-05 | 0.3856 | 0.6999 |
| 1 | C | rs6663565 | 41232781 | *SCMH1* | 1599 | 0.05555 | 0.03581 | 0.001504 | 1.551 | 0.121 |
| 1 | C | rs17038164 | 118574711 | *SPAG17* | 1599 | -0.0576 | 0.04009 | 0.001291 | -1.437 | 0.151 |
| 1 | G | rs11205277 | 146705945 | Histone class 2A, *MTMR11, SV2A, SF3B4* | 1599 | 0.05358 | 0.03564 | 0.001413 | 1.503 | 0.133 |
| 1 | G | rs678962 | 168921546 | *DNM3* | 1596 | -0.03139 | 0.04493 | 0.0003062 | -0.6987 | 0.4848 |
| 1 | A | rs2274432 | 180752602 | *C1orf19,GLT25D2* | 1554 | 0.05121 | 0.03936 | 0.00109 | 1.301 | 0.1934 |
| 1 | A | rs3942992 | 224079131 | *ZNF678* | 1599 | -0.01098 | 0.04696 | 3.43E-05 | -0.2339 | 0.8151 |
| 2 | G | rs3791679 | 56008543 | *EFEMP1, PNPT1* | 1596 | -0.1669 | 0.04079 | 0.0104 | -4.092 | 4.49E-05 |
| 2 | T | rs1052483 | 219759853 | *IHH, CRYBA2, FEV, SLC23A3, TUBA1* | 1583 | -0.06978 | 0.06188 | 0.0008038 | -1.128 | 0.2596 |
| 3 | C | rs9841212 | 135674636 | *ANAPC13, CEP63* | 1595 | -0.06289 | 0.03779 | 0.001735 | -1.664 | 0.09629 |
| 3 | A | rs6763931 | 142585531 | *ZBTB38* | 1596 | 0.05824 | 0.03588 | 0.001651 | 1.623 | 0.1047 |
| 4 | T | rs6842303 | 17530324 | *LCORL, NCAPG* | 1597 | 0.03162 | 0.04098 | 0.000373 | 0.7715 | 0.4405 |
| 4 | C | rs6830062 | 17693999 | *LCORL, NCAPG* | 1599 | 0.01247 | 0.04678 | 4.45E-05 | 0.2665 | 0.7899 |
| 4 | A | rs1812175 | 145932449 | *HHIP* | 1597 | -0.09088 | 0.04689 | 0.00235 | -1.938 | 0.05275 |
| 5 | T | rs10472828 | 32924575 | *NPR3* | 1599 | 0.007703 | 0.03598 | 2.87E-05 | 0.2141 | 0.8305 |
| 6 | A | rs12198986 | 7665058 | *BMP6* | 1598 | 0.02092 | 0.03588 | 0.0002129 | 0.5829 | 0.56 |
| 6 | G | rs10946808 | 26341366 | Histone class 1, Butyrophilin genes | 1595 | -0.08184 | 0.03855 | 0.002821 | -2.123 | 0.03392 |
| 6 | C | rs2844479 | 31680935 | HLA class III | 1599 | -0.04648 | 0.03594 | 0.001046 | -1.293 | 0.1961 |
| 6 | G | rs3130050 | 31726740 | HLA class III | 1597 | 0.1365 | 0.05299 | 0.004141 | 2.575 | 0.0101 |
| 6 | T | rs185819 | 32158045 | HLA class III | 1596 | 0.1285 | 0.03506 | 0.008354 | 3.664 | 0.000256 |
| 6 | G | rs1776897 | 34302989 | *HMGA1, LBH* | 1598 | 0.04851 | 0.06026 | 0.0004059 | 0.8051 | 0.4209 |
| 6 | A | rs2814993 | 34726871 | *C6orf106* | 1576 | 0.144 | 0.0539 | 0.004515 | 2.672 | 0.007624 |
| 6 | A | rs4713858 | 35510763 | *ANKS1, TCP11, ZNF76, DEF6, SCUBE3* | 1599 | -0.1081 | 0.04867 | 0.003081 | -2.221 | 0.02646 |
| 6 | C | rs314263 | 105499438 | *LIN28B, HACE1, BVES, POPDC3* | 1599 | 0.01874 | 0.03897 | 0.0001448 | 0.4809 | 0.6306 |
| 6 | T | rs1490388 | 126877348 | *C6orf173 / LOC387103* | 1596 | 0.03024 | 0.03512 | 0.0004648 | 0.8609 | 0.3894 |
| 6 | G | rs3748069 | 142809326 | *GPR126* | 1599 | -0.07657 | 0.0374 | 0.002617 | -2.047 | 0.04081 |
| 7 | T | rs798544 | 2536343 | *GNA12* | 1599 | 0.006449 | 0.0399 | 1.64E-05 | 0.1617 | 0.8716 |
| 7 | C | rs1182188 | 2643226 | *GNA12* | 1599 | 0.00743 | 0.04002 | 2.16E-05 | 0.1857 | 0.8527 |
| 7 | A | rs849141 | 27958331 | *JAZF1* | 1599 | 0.02939 | 0.03974 | 0.0003423 | 0.7395 | 0.4597 |
| 7 | C | rs2282978 | 91909061 | *CDK6, PEX1, GATAD1, ERVWE1* | 1599 | 0.01004 | 0.03695 | 4.62E-05 | 0.2718 | 0.7858 |
| 7 | C | rs11765954 | 91925346 | *CDK6, PEX1, GATAD1, ERVWE1* | 1599 | 0.01451 | 0.03907 | 8.63E-05 | 0.3714 | 0.7104 |
| 8 | C | rs10958476 | 57258362 | *PLAG1, MOS, CHCHD7, RDHE2, RPS20, LYN, TGS1, PENK* | 1594 | 0.1362 | 0.04428 | 0.005907 | 3.076 | 0.002136 |
| 8 | C | rs7846385 | 78322734 | *PXMP3, ZFHX4* | 1599 | -0.03217 | 0.03952 | 0.0004148 | -0.8141 | 0.4157 |
| 9 | G | rs4448343 | 95345925 | *PTCH1* | 1599 | 0.04858 | 0.03733 | 0.001059 | 1.301 | 0.1933 |
| 9 | A | rs4743034 | 106711908 | *ZNF462* | 1599 | 0.03034 | 0.04271 | 0.000316 | 0.7105 | 0.4775 |
| 12 | C | rs8756 | 64646019 | *HMGA2* | 1599 | 0.05093 | 0.03507 | 0.001319 | 1.452 | 0.1466 |
| 12 | G | rs3825199 | 92479422 | *SOCS2, MRPL42, CRADD, UBE2N* | 1599 | 0.08657 | 0.04438 | 0.002376 | 1.95 | 0.0513 |
| 13 | C | rs1239947 | 50004556 | *DLEU7* | 1598 | 0.07469 | 0.03802 | 0.002412 | 1.965 | 0.04964 |
| 14 | C | rs910316 | 74695795 | *TMED10* | 1599 | -0.005116 | 0.03509 | 1.33E-05 | -0.1458 | 0.8841 |
| 14 | C | rs7153027 | 91496975 | *TRIP11, FBLN5, ATXN3, CPSF2* | 1592 | -0.02704 | 0.03584 | 0.0003578 | -0.7544 | 0.4507 |
| 15 | C | rs2554380 | 82106888 | *ADAMTSL3, SH3GL3* | 1579 | 0.02203 | 0.04665 | 0.0001415 | 0.4724 | 0.6367 |
| 15 | T | rs11633371 | 87157836 | *ACAN* | 1599 | 0.07099 | 0.03545 | 0.002505 | 2.003 | 0.04538 |
| 15 | A | rs4533267 | 98603794 | *ADAMTS17* | 1599 | 0.02824 | 0.03899 | 0.0003282 | 0.7241 | 0.4691 |
| 17 | A | rs3760318 | 26271841 | *CRLF3, ATAD5, CENTA2, RNF135* | 1599 | -0.04332 | 0.03586 | 0.0009127 | -1.208 | 0.2273 |
| 17 | A | rs4794665 | 52205328 | *NOG, DGKE, TRIM25, COIL, RISK* | 1599 | -0.003459 | 0.03513 | 6.07E-06 | -0.09848 | 0.9216 |
| 17 | A | rs757608 | 56852059 | *BCAS3, NACA2, TBX2, TBX4* | 1585 | 0.03449 | 0.03812 | 0.0005169 | 0.9048 | 0.3657 |
| 18 | G | rs4800148 | 18978326 | *CABLES1, RBBP8, C18orf45* | 1599 | -0.06726 | 0.04344 | 0.001499 | -1.548 | 0.1217 |
| 18 | T | rs530550 | 45105636 | *DYM* | 1599 | -0.05102 | 0.03612 | 0.001248 | -1.412 | 0.158 |
| 19 | G | rs12459350 | 2127586 | *DOT1L* | 1597 | 0.0009728 | 0.03564 | 4.67E-07 | 0.02729 | 0.9782 |
| 20 | A | rs967417 | 6568893 | *BMP2* | 1599 | 0.02263 | 0.03556 | 0.0002535 | 0.6363 | 0.5246 |
| 20 | C | rs4911494 | 33435328 | *UQCC, GDF5, CEP250, EIF6, MMP24* | 1598 | 0.1064 | 0.03746 | 0.00503 | 2.841 | 0.00456 |

NMISS: number of individuals tested; BETA: regression coefficient for the test SNP; SE: standard error of the regression coefficient; R2: r^2^ value in linear regression; T: test statistic; P: two-sided trend test *P*-value. The direction of effect is shown for the minor allele in each case.
